# Answering the missed call: Initial exploration of cognitive and electrophysiological changes associated with smartphone use and abuse

**DOI:** 10.1371/journal.pone.0180094

**Published:** 2017-07-05

**Authors:** Aviad Hadar, Itay Hadas, Avi Lazarovits, Uri Alyagon, Daniel Eliraz, Abraham Zangen

**Affiliations:** Department of Life Science and the Zlotowski Centre for Neuroscience, Ben-Gurion University, Beer-Sheva, Israel; Ariel University, ISRAEL

## Abstract

**Background:**

Smartphone usage is now integral to human behavior. Recent studies associate extensive usage with a range of debilitating effects. We sought to determine whether excessive usage is accompanied by measurable neural, cognitive and behavioral changes.

**Method:**

Subjects lacking previous experience with smartphones (n = 35) were compared to a matched group of heavy smartphone users (n = 16) on numerous behavioral and electrophysiological measures recorded using electroencephalogram (EEG) combined with transcranial magnetic stimulation (TMS) over the right prefrontal cortex (rPFC). In a second longitudinal intervention, a randomly selected sample of the original non-users received smartphones for 3 months while the others served as controls. All measurements were repeated following this intervention.

**Results:**

Heavy users showed increased impulsivity, hyperactivity and negative social concern. We also found reduced early TMS evoked potentials in the rPFC of this group, which correlated with severity of self-reported inattention problems. Heavy users also obtained lower accuracy rates than nonusers in a numerical processing. Critically, the second part of the experiment revealed that both the numerical processing and social cognition domains are causally linked to smartphone usage.

**Conclusion:**

Heavy usage was found to be associated with impaired attention, reduced numerical processing capacity, changes in social cognition, and reduced right prefrontal cortex (rPFC) excitability. Memory impairments were not detected. Novel usage over short period induced a significant reduction in numerical processing capacity and changes in social cognition.

## Introduction

Many human functions are now supported and mediated by smartphone devices. It has been argued that usage comes with a price: it has been associated with an increasing accident rate, sleep disorders, undesirable mental health outcomes and persistent changes in behavior and personality [1,2]. Indeed, the potential negative impact of smartphone on human behavior has not completely escaped the eyes of policy makers. For instance, authorities began imposing heavy penalties on drivers and even on pedestrians using smartphones [3–5].

Given the sheer frequency and duration of daily smartphone usage it is conceivable that changes in cognition, behavior and psychological states may be observed in users. Such changes have been found in intensive users who present an exaggerated and debilitating usage [6,7]. Recent studies showed an association between such usage and impulsive behaviors [8], pathological dependence [9], low emotional stability and self-esteem [10], chronic stress [11], depression [12,13], and sleep disturbances [2,11]. A larger study of 5051 adolescences, found that heavy users were significantly more likely to attempt suicide as compared with normal users [12]. However, these recent studies offer primarily questionnaires-based correlative data and thus little can be inferred in terms of causal changes to cognition and behavior.

Some researchers conceptualized behavioral changes associated with smartphones and similar technologies as a behavioral addiction [14–16]. Kown et al. (2013) generated and validated the Smartphone Addiction Scale (SAS), based on previous internet addiction diagnostics, and several similar scales have been developed [17]. However, despite the upsurge in diagnostic instruments, to date there is no evidence demonstrating that smartphones alter objectively measured behavior [18]. In order to bridge this gap in the literature this multifaceted study sought to explore smartphone associated changes on 3 planes: 1) self-reported and objective records of behavioral tendencies, 2) behavioral task performance, and 3) recording of a relevant neural circuitry.

The first was addressed by using series of questionnaires assessing traits and behavioral tendencies that characterize problematic use of mobile phones and internet (11,12,16). Specifically, psychosocial disorders, depressive symptoms, and loss of impulse control emerged from the literature as dominant in such users [19–21]. For instance, internet usage has been shown to increase impulsivity in heavy users, and similar results were obtained in computer gaming addiction [21]. We thus predicted that heavy smartphone users will present with abnormal scores of social cognitions, depression, and ADHD measures. To account for the versatile nature of self-report data we also monitored actual smartphone usage using a smartphone software. We sought to assess whether objectively measured usage will be proportionally linked to severity of reported symptoms.

The second was addressed by employing tasks assessing impulsivity, attention in information processing and memory. The selection of these domains stemmed from the current knowledge in the fields on internet addiction disorder (IAD). The most consistent behavioural finding in these disorders is poor inhibitory control suggesting greater impulsivity [22,23]. Equally important, several studies suggested an association of attention grabbing stimuli with memory and attention deficits [24–26]. In addition, numerous studies implicated digital addictions (internet and gaming) in more generalized executive control dysfunctions [27,28]. Executive control processes such as working memory performance and decision making were reported to be particularly reduced in the presence of digital devices. We thus selected a classic response inhibition task [29], a speeded and difficult numerical information processing task, and a recognition task. We speculated that given the overarching dependence of smartphone users on the devices for simple calculations and as a memory aid, they may exhibit impaired capacity for *technologically unassisted* information processing and retention of information.

The third plane, relevant neural activity, was addressed by measuring right prefrontal cortex (rPFC) excitability previously implicated in impulse inhibition [30]. Internet and gaming addiction, currently defined as impulse control disorder [24], have both been associated with abnormal prefrontal activity at rest [24]. In a recent review by Weinstein and colleagues the neural mechanisms underlying internet and gaming addiction have been suggested to closely resemble the neuropathology of substance addiction [31]. Reduced gray and white matter volume have been repeatedly found in the prefrontal cortex, the orbitofrontal cortex and the supplementary motor area of subjects with internet and gaming addiction; changes that correlated with the duration of the disorder [24] [31]. We thus measured right prefrontal activity to establish whether sensitive neuronal changes occur in association with heavy smartphone usage. For this purpose, we measured several indices of rPFC excitability. First, early transcranial magnetic stimulation (TMS) evoked potential (TEP) was used to compare glutamatergic-related [32] cortical excitability at rest. Second, long interval cortical inhibiton (LICI) was used to evaluate a correlate of gama-aminobutyric acid (GABAb)- mediated activity of inhibitory interneurons [33,34].

**Fig 1:**
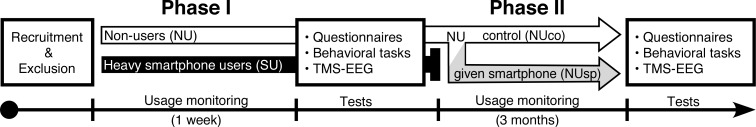
Timeline of recruitment, allocation and procedures. 35 participants (19 females; mean age 25±3.8 years [range: 21–32 years]) lacking any previous experience with or possession of a smartphone, but in possession of a standard mobile phone, were allocated to the nonusers (NU) group. The second, heavy smartphone users (SU) group, was recruited using the smartphone addiction scale (SAS) and the mobile phone involvement questionnaire (MPIQ) administered online to 2711 participants. Participants who scored more than 2 standard deviations above the mean in both questionnaires were recruited via telephone interview to form the SU group. This group consisted of 16 participants (9 females; mean age 24±2.5 years [range: 21–27 years]). A subset (n = 28 total; n = 25 with TMS) of the NU participants consented to a second phase. In the second phase 11 of the NU participants (7 females; mean age 24.7±2 years [range: 21–29]) were randomly selected to receive smartphone devices for a 3 months period (NUsp) while 14 others of the NU participants (8 females; mean age 24.9±2.2 years [range 22–28]) served as matched controls (NUco), the remainder of the NU sample and the SU group were released from the study immediately after the first phase. See S1 and S2 Tables for full demographics of all groups.

Finally, we sought to determine whether smartphone usage can autonomously *cause* such changes. For this purpose we employed an additional longitudinal randomized controlled design in which a sample of nonusers received a smartphone while a matched control sample remained with their original mobile phone (Fig 1); a design that serves two aims. First, it allows identification of cognitive, behavioral and neuronal alterations in heavy smartphone users, by comparing the results of the two populations (heavy- vs. non-users). Second, it allows to determine whether usage produces measurable behavioral and neural changes.

## Methods

### Participants

Fig 1 presents the timeline and allocation of the groups to the different study phases (note color coding). Participants in the NU group were recruited using advertisements in university campus and participants in the SU group were selected using a questionnaire [15, 35] as detailed in Fig 1. Overall 60 participants were selected and 51 participated in the study between June 2013- December 2014 (9 of the selected participants refused TMS measurements). Participants were screened for safety contraindications for TMS, psychiatric diagnosis and learning impairments. Overall four groups were tested. Two groups were tested in the first phase; heavy smartphone users (SU, n = 16) and nonusers (NU, n = 35). A subset (n = 28 total; n = 25 with TMS) of the NU participants consented to a second phase. These were randomly selected to either receive smartphone devices for a 3 months period (NUsp, n = 12, 1 refusing TMS) or serve as controls (NUco, n = 16, 2 refusing TMS). The reminder of the sample and the SU group was released from the study at this point.

The experimental procedures were approved by and in accordance with the local Helsinki Committee of Ben Gurion University. All participants provided written informed consent prior to the study. Participants received 150 NIS (45$) payment for each of the two experimental phases.

### Behavioral measurements

#### Smartphone application

The application App Manager Pro 2 (ATM2 Ltd, 2013) was installed on participants’ smartphones following the first study phase (i.e. for SU and NUsp groups). App data was recorded for seven days of usage and downloaded for offline analysis. The software recorded the duration and frequency of usage for each application on the phone.

#### Speeded numerical processing task

Subjects sat 50 cm in front of a 19-inch CRT monitor. Each trial was briefly presented for a duration of 2 seconds. The task centrally presented a rapidly changing series of arithmetic problems (e.g. 4–6+3 =?) each for 600 milliseconds in white on a black screen. Next, the following question appeared ‘greater or smaller than 4?’ to which participants were instructed to respond as quickly as possible by pressing the left or right mouse button before the onset of the following stimulus.

#### Memory task

Subjects were presented with an array of eight simple geometric patterns and were required to remember their orientation. Immediately following, a recognition test was conducted. It presented four possible alternatives, each depicting one of the previously shown patterns in a different orientation. Participants used the keyboard number pad to indicate which of the four alternatives matches the previously presented pattern.

#### Stop signal task

A modified version of the visual stop signal task was presented on a lab PC using Eprime (PST Inc., E-Prime 2.0). The task presented two visual cues (‘x’, ‘o’) to which participants had to respond by pressing one of two corresponding buttons using either their left or right index fingers. In 25% of trials a stop signal was shown (white square) immediately after the visual cue to which subjects had to withhold their response. The stop signal delay (SSD) from visual cue onset changed in a staircase dynamic-tracking manner, depending on performance. A detailed description appears in Berger et al. (2013) [29].

#### Questionnaires

The Hebrew version of the following questionnaires were administered: Beck Depression Inventory (BDI) (30), Conners’ Adult ADHD Rating Scales (CAARS) [36], Revised Self-Monitoring Scale (RSMS) and the Concern for Appropriateness Scale (CAS) [37].

In addition to the ADHD questionnaire we assessed impulsivity using the monetary delay discount task [38]. In this task participants were asked to decide between small immediate monetary rewards to larger delayed rewards. Participants were told that one of their choices will be monetized at a random selection.

### TMS-EEG protocol

Electroencephalogram (EEG) activity was recorded both during the Stop Signal task and during TMS using a 64 electrodes Waveguard cap and a TMS compatible EEG amplifier referenced to Cz electrode. Data were acquired using ASA™ version 4.7.3 (ANT neuro, Enschede, Netherlands). Impedance was kept below 10 kOhm. PO6 served as a ground electrode. Recording frequency was set to 2048 Hz, and digitized with a 24-bit AD converter.

Pulses were applied after EEG set up over the cap using a 70 mm figure-of-eight coil (external casing diameter ~90 mm for each loop) connected to a MagstimRapid2 biphasic stimulator (Magstim Co., Whitland, Carmarthenshire, U.K.). Pulses were delivered at 120% of RMT above the rPFC (detailed in SI). The stimulation protocol consisted of a series of 5 single pulse followed by a series of 5 paired pulses at 0.2Hz. Paired pulses were interleaved by 100 ms between the conditioning pulse/stimulus (CS) and the test pulse (TS) to induce LICI [39]). This series was repeated 10 times cumulating at 50 single and 50 paired pulses. A post- report form was used to document any adverse effects of TMS [40].

### Session procedure

Subjects completed the computerized tasks and questionnaires and then seated in the TMS-EEG lab. Following EEG setup subjects performed the Stop Signal Task (see S1 File). Then, RMT was determined and TMS coil was locked in position (see S1 File) and TMS was administered.

NU subjects were asked whether they would like to continue to the second phase. Consenting subjects were randomly selected to either receive a smartphones (Galaxy 2, Samsung) installed with ATM2 or to remain with their simple phones. 90 days following this intervention the above procedure was repeated for both exposure (NUsp) and control (NUco) groups (Fig 1).

### Data processing

Electrophysiological data during TMS was processed offline using EEGlab toolbox for Matlab. EEG data during Stop Signal task is not reported given the null behavioral results (see Results) (detailed data preprocessing in S1 File).

Two measures based on TMS evoked potential (TEP) were calculated: The first measure, named early TEP, represents the immediate activity evoked by the TMS. This measure was calculated as the rectified average amplitude 15–40 ms [41,42] after a single pulse, of all electrode positioned under the center of the TMS coil (FC4, FC6, F4, F6). The second TMS-based measure calculated was LICI taken from the same electrodes [33,39,43]. One obstacle in comparing single and paired TEP is the artefact of the late activity evoked by the CS on the early evoked response of the TS. In order to overcome this obstacle, we used the method offered by Daskalakis et al. [39], wherein for each subject the average cortical evoked potential elicited by a single pulse was shifted by 100 ms and subtracted from the average cortical-evoked potentials elicited by the TS of the paired pulse. LICI was then calculated as the ratio between the area under the rectified curve (AUC) of the ERP following paired pulses with the AUC of the ERP following single pulse, using the following equation (Eq 1) [39]:

$$\mathrm{LICI}=\left[1-\frac{\mathrm{rectified}\;\mathrm{AUC}\;\left(\mathrm{paired}\right)}{\mathrm{rectified}\;\mathrm{AUC}\;\left(\mathrm{single}\right)}\right]*100$$ Eq 1

The time window of this calculation was based on inspection of grand average and reported timing for LICI activity in the literature [44].

### Statistical analysis

Phase 1 data sets (SU vs. NU) were compared using t-tests. Phase 2 data (NUco and NUsp at baseline and after intervention) was submitted to a 2X2 mixed design ANOVA crossing the between subjects factor of Manipulation (Control vs Exposure) and the within subjects factor of Time (Baseline vs 3 Months). All tests were two tailed run at alpha level of 0.05. Significance levels were adjusted using a Bonferroni procedure in all instances of multiple comparisons (i.e., CAARS six subscales comparisons and correlation of app usage with the various subscales) and Fisher Exact tests were employed for post-hoc analyses. In all measures, data greater than 2 standard deviations from group mean was excluded (for detailed statistics see figure legends, for full data see https://zenodo.org/record/810695).

## Results

### Demographics

Socio-demographics characteristics of participants in phase 1 and 2 are shown in Fig 1, S1 and S2 Tables. No significant differences were observed between groups.

### Questionnaires

**Fig 2:**
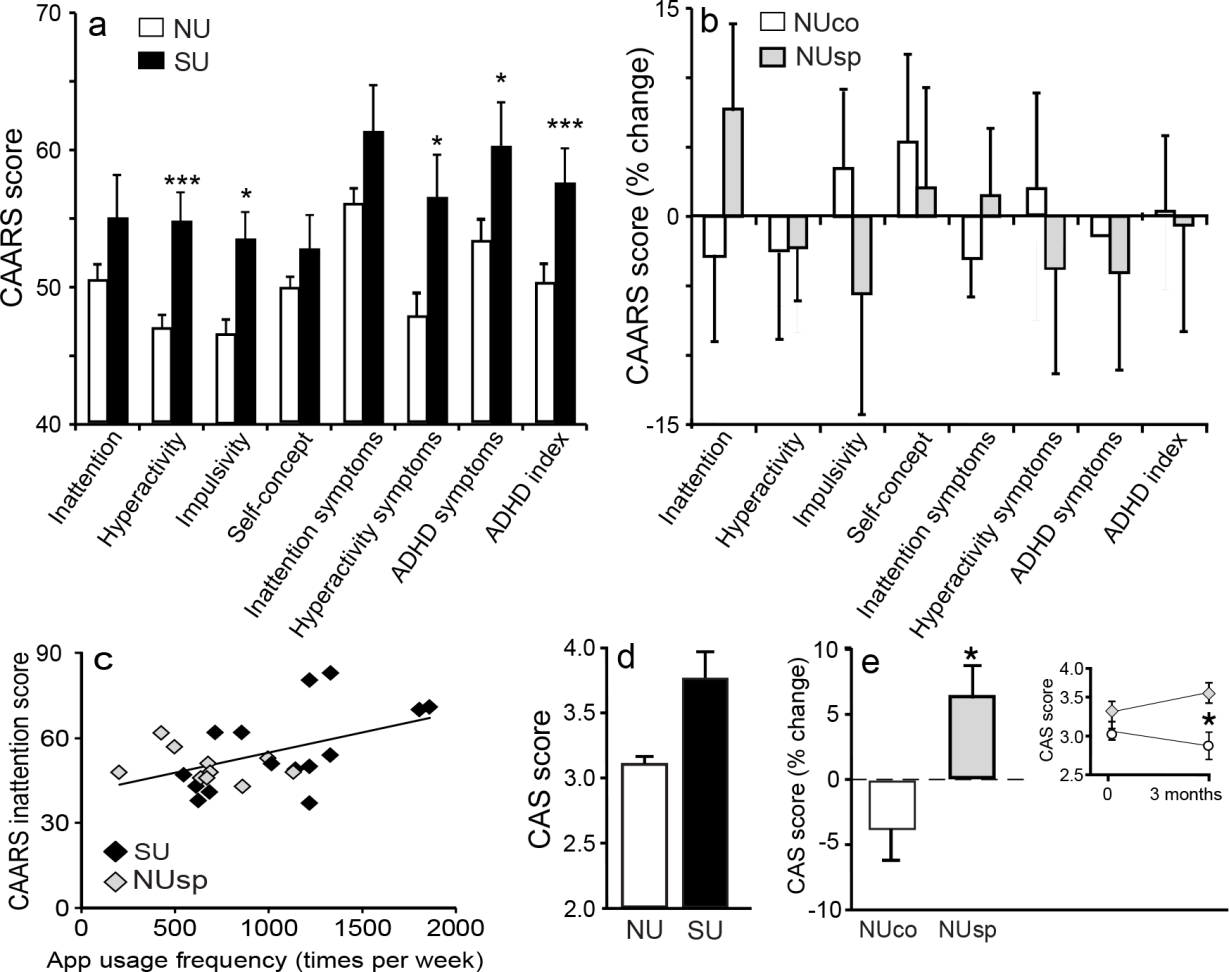
Questionnaire results and their correlation with real usage frequency. Bar charts show mean ± SEM values. (a) CAARS score from the first phase. Total ADHD index (t (49) = 2.8, p = 0.008, d = 0.8), impulsivity (t (49) = 2.5, p = 0.01; p_corrected_ = 0.07, d = 0.75) and hyperactivity (t (49 = 2.9, p = 0.006, p_corrected_ = 0.035, d = 0.7) scores were significantly higher in the SU group as compared to the NU group. ADHD symptoms (t (49) = 2.2, p = 0.03, p_corrected_ = 0.21, d = 0.61) and hyperactivity symptoms (t (49) = 2.3, p = 0.02, p_corrected_ = 0.14, d = 0.68) also showed a non-significant trend in the same direction (b) Changes in CAARS scores between end and start of intervention in the second study phase is shown. No significant differences between groups were observed (p>0.2 for all domains). (c) The scatterplot shows a marginally significant positive correlation between the total frequency of app usage and CAARS inattention subscales of both SU and NUsp participants (R (24) = 0.5, p = 0.013, p_corrected_ = 0.08). (d) SU obtained higher CAS (social concern) score than NU (t (49) = -2, p = 0.052, d = 0.61). (e) A significant interaction effect of the manipulation on CAS scores was found (F (1,24) = 6.4, p = 0.018, η^2^_p_ = 0.21). Posthoc analysis revealed a significant increase (p = 0.034) from baseline in CAS score in the NUsp group while NUco showed a mild non- significant decrease in CAS (p = 0.15). p_corrected_ term refers to instances of multiple comparisons where the p value was Bonferroni corrected.

Attentional deficiencies, as shown in the CAARS questionnaire, were significantly higher in SU than those reported in NU (Fig 2A). In the second phase, a non-significant increase in inattention was observed after 3 months of smartphone exposure in the NUsp group, but not in the NUco group (Fig 2B).

Higher social concern (CAS) was reported by the SU group as compared to NU group (Fig 2D). In the second phase, a significant Manipulation X Time interaction effect was found. Post-hoc test confirmed that NUsp suffered a significant increase in CAS while NUco did not show such pattern (Fig 2E).

No significant group differences were found in BDI and RSMS.

### Smartphone usage data

As expected, app data showed that SU participants used their smartphones more frequently than NUsp (SU 1068±104; NUsp 678±105; t(23) = 2.6, p = 0.016, d = 1.1). App Timer Mini 2 (ATM2) data was correlated to questionnaires showing significant group effects (i.e. CARRS and CAS). A positive correlation was found between frequency of smartphone usage and inattention (Fig 2C).

### Behavioral tasks

**Fig 3:**
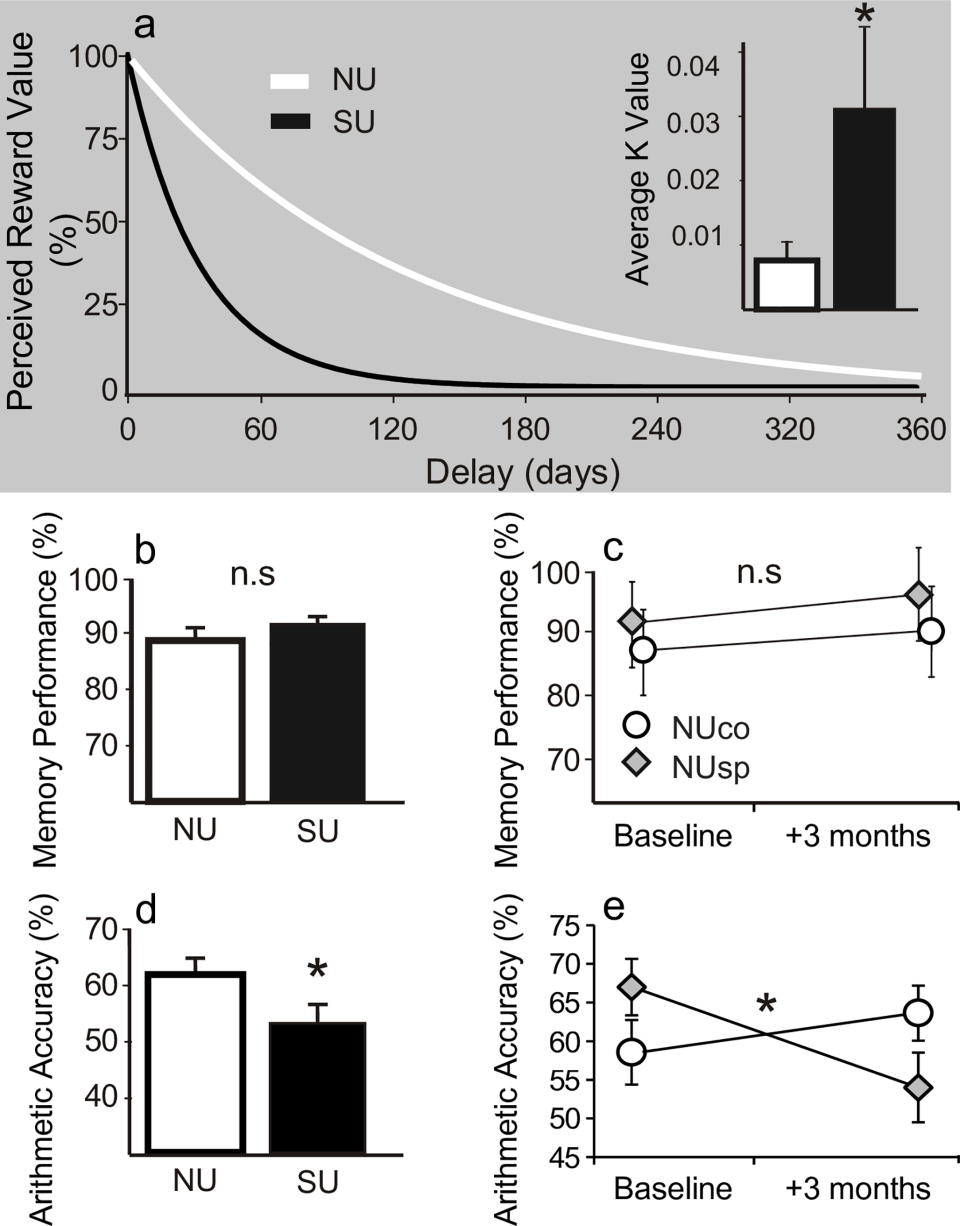
Bar charts show mean ± SEM values of main behavioural tasks. (a) Mean K coefficient values were significantly higher in SU than in NU (t (49) = 2.14, p = 0.04, d = 0.5) and the resulting discounting curve demonstrated a markedly steeper discounting rate. This effect was not observed in the second phase (F(1,23) = 0.7, p = 0.4). (b) Mean memory accuracy did not differ between SU and NU groups (t(45) = 0.6, p = 0.5). (c) No significant effect on memory was observed (p = 0.6) (d) SU showed significantly poorer arithmetic accuracy as compared to NU (t(42) = 2, p = 0.05, d = 0.6; 6 participants scored zero on accuracy indicating less than 3 accurate and timely trials and were discarded from the analysis). (e) A significant Manipulation X Time interaction effect was found [F (1,22) = 5.3, p = 0.03, η^2^_p_ = 0.19)]. Posthoc analysis highlighted a significant (NUsp group; p = 0.025) decline from baseline in accuracy levels of smartphone users while such pattern was not found in the control group (NUco group; n.s).

For the delayed discounting task, K coefficients (42; 50) values were obtained and implemented in the hyperbolic discounting function (Fig 3A). On average, the SU group had significantly higher K coefficient value than that of the NU group and so the resulting discounting curve demonstrated a markedly steeper discounting rate for the SU group. Such effect was not observed in the second phase, indicating that 3 months of smartphone usage did not induce a significant change in performance.

In the short-term non-verbal memory task, no significant differences were observed between the groups (Fig 3B), and 3 months of smartphone use by novel users did not induce any effects (Fig 3C).

In the speeded arithmetic task, heavy users were found to be significantly less accurate (Fig 3D), while response times (RT) did not vary (p = 0.4). This effect was corroborated by a significant Manipulation X Time interaction effect found in the second phase: 3 months of smartphone use induced a significant reduction in accuracy in the arithmetic task (Fig 3E). No significant RT effects were found.

Stop Signal task parameters were virtually identical across groups. Stop Signal RT (SU, 239±32 ms; NU, 241±41 ms, t(47) = 0.2, p = 0.9), mean RT (SU, 583±75 ms; NU, 601±114 ms, t(47) = 0.2, p = 0.9, t(47) = 0.6, p = 0.5) and Go Errors (SU, 9.1±6.2%; NU, 4.37±3.9%, t(47) = 1, p = 0.3) showed no significant effects. No significant effects were observed in the second phase.

### Electrophysiological measures

**Fig 4:**
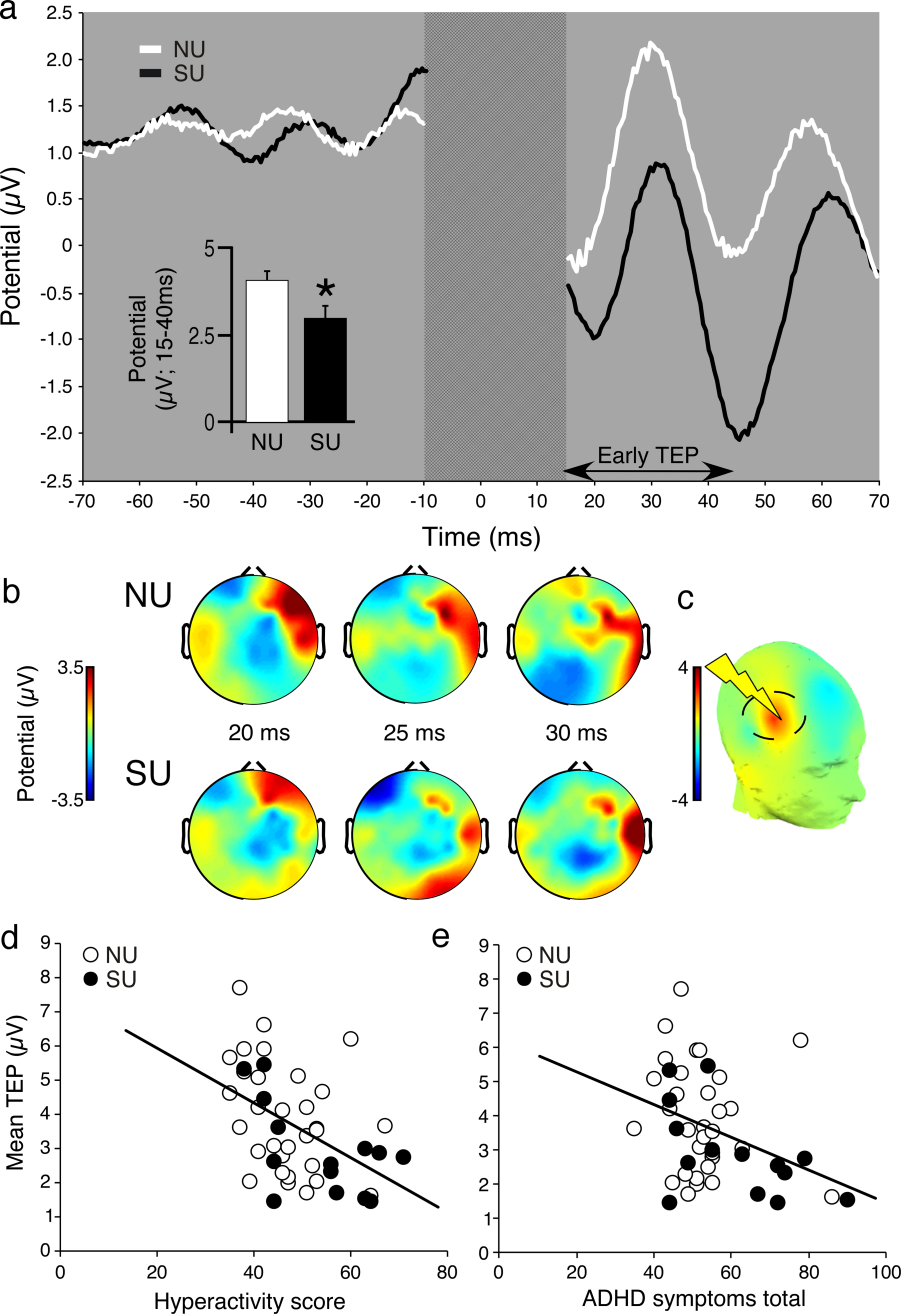
Electrophysiological responses to single TMS pulses in the rPFC. (a) Grand average rectified ERP plots of early TEP taken from all electrodes under the stimulation coil (FC4, F4, FC6, F6) in the Smartphone users (SU) and nonusers (NU) groups. On the top right, bar charts represent mean ± SEM mean area under curve (AUC) of average early TEP taken from (t (45) = 2.4, p = 0.03, d = 0.6). For a longer time window see S1 Fig. (b) Two dimensional topographical plots of EEG recorded activity at 20 ms, 25 ms and 30 ms after pulse onset for SU and NU groups. (c) Three-dimensional topographical plot of group TEP difference (Δ plot) representing the difference in average voltage between SU and NU participants over the time-period 15–40 ms. (d) Early TEP was showed significant negative correlation with hyperactivity subscale as measured in the CAARS questionnaire (R (45) = -0.49, p = 0.006, Bonferroni corrected) (e) The magnitude of the early TEP was negatively correlated with total ADHD symptoms phase 1 (R (45) = -0.39, p = 0.013).

Early TEP (15–40 ms) induced by single TMS pulses over the rPFC of SU participants was significantly lower than that of NU participants (Fig 4). However, this measure was not affected by 3 months of smartphone usage in novel users. While ANOVA revealed no Exposure X Time interaction effect, a mild (and non-significant) increase in average early TEP was observed in both NUco and NUsp groups.

Early TEP data from the first phase was correlated with questionnaire data showing significant effects (i.e. CAARS and CAS). A negative correlation was found between mean early TEP and ADHD total symptoms (as well as hyperactivity subscale), indicating a relation between reduced rPFC excitability and attentional deficits and hyperactivity (Fig 4D and 4E).

LICI was established in all groups (S1 Fig). Put differently, grand-averaged TEP produced by paired TMS pulse was significantly reduced as compared to single pulse alone in all groups (mean LICI across groups 27.3±5.1%). This pattern confirmed that the stimulation protocol successfully induced neuronal inhibition in the stimulated cortical area. However, no significant group effects were found in both study phases (S1 Fig).

## Discussion

The present study suggests that extensive usage of smartphone devices may be associated with deficits in cognitive capacities, social attitudes and rPFC excitability. Moreover, 3 months of smartphone usage by previously smartphone naïve subjects causes a significant reduction in arithmetic accuracy and increased social concern. This is the first report of an attempt to systematically manipulate exposure to smartphones and measure consequent behavioral and neural changes.

### Attention

The most consistent behavioral effect found in the current study, which was repeated in both phases of the study, was a marginal decrease in numerical processing capacity. Heavy smartphone users were found to be significantly less accurate than their matched nonuser pairs on a simple speeded arithmetic task. Furthermore, participants receiving smartphones suffered a significant decrease in accuracy levels after 3 months of usage. The results obtained by the control group removes the possibility of an external confound. Moreover, the absence of RT differences reduce the likelihood of differential task strategies employed by the groups as they would have led to apparent speed accuracy tradeoffs.

The numerical processing deficits reported here may be feasibility associated with several cognitive and neural mechanisms [45]. First, impairments in working memory capacity might have driven poor accuracy rates. However, given the relative low memory load required by the task (adding up 3 single digit numbers) it seems less plausible that such deficits exist in a sample of highly functioning young adults [46]. Indeed, we found no memory impairments. Second, differences in motivation could also explain this effect. Reduced motivation has been previously reported in heavy internet and computer games users [24]. However, given that other motivationally demanding tasks, such as the Stop Signal task, showed no groups differences, this explanation appears insufficient. Third, numerical representation and the ability to store and manipulate such information, which has been previously suggested to involved distinct activity of the intraparietal sulcus [47], may be specifically impaired in smartphone users. Speculatively, the overreliance upon the device for numerical calculation and the absence of device-free arithmetic calculation might have rapidly eroded this mental capability, as the popular heuristic for neural plasticity suggests ‘use it or loss it’. Although we cannot rule out this intuitive explanation we currently have no supportive data for it.

We thus suggest a forth account that emphasizes the role of reduced attentional capacity in driving the deficits in arithmetic capacity. Earlier studies conducted on heavy smartphone users reported difficulties in sustaining attention and reduced sleep quality and attentional deficiencies are consistently reported in IAD 11,2 [48]. Indeed, merely 3 months of usage induced a trend for increased inattention (Fig 2B) and it positively correlated with frequency of smartphone usage (Fig 2D). We therefore suggest that a major psychological mechanism behind these smartphone-induced changes is diminished capacity to sustain attention.

### Social cognition

Our measure of social concern also showed consistent changes in both study phases. Negative preoccupation with social representations measured by CAS was higher in SU than in NU. Most critically, those NU participants who later received smartphones suffered a significant increase in social concern. No such changes were observed in the RSMS scores, which measures the degree of psychologically healthy active and flexible social approach. This dissociation suggests that usage fosters the development of a particular anxiety about self-presentation but does not necessarily hinders other positive social attitudes.

High CAS score reflects increased fear of social threats leading to high conformity and anxiety regarding social acceptance and approval. It has been associated with a greater tendency to hostility and aggression [37]. Unfortunately, we were unable to analyze the type of smartphone activity due to excessive usage of the smartphone’s internet browser which masks which websites/functions are accessed. Hence we can only speculate that this change in social cognition results from the constant engagement with social media platforms, text messages and calls. Indeed under circumstances of extensive usage the device might effectively serve as a social monitoring agent to which the user need to provide continuous feedback within a framework of agreed and acceptable social behavior [49]. Furthermore, by providing immediate informational rewards and clear signs of social acceptance/rejection, the device encourages a state of constant preoccupation with self-presentation. Nonetheless, we acknowledge the young age of our sample (Fig 1) and aware that this effect may be less pronounced in older population.

### Impulsivity

Both subjective introspective and objective behavioral measure demonstrated that SU were more impulsive than NU participants. Heavy users scored significantly higher on scales of impulsivity and users were found significantly more likely to discount the value of money rewards in the presence of delay. The high K coefficient values obtained in the delay discounting task reflect impulsive decision making and biased perception of the reward value due to an increased urge for immediate gratification [38]. However, unlike the alterations found in numerical processing or social cognition, 3 months of smartphone usage did not induce significant changes in impulsivity. This pattern of results is consistent with a previous report on internet and gaming addiction where a single session of internet usage resulted in increased impulsivity in individuals with high but not low levels of self-reported problematic internet behaviours [21]. We cannot estimate, however, whether our screening procedure selected heavy users which were initially impulsive, or whether it selected a group of heavy users which turned impulsive due the intensive usage. Furthermore, we found no evidence for deficits in behavioral inhibition as measured in the Stop Signal task. Hence, our finding is less conclusive with regard to the effect of smartphone usage on delay discounting and impulsivity.

### Early TMS evoked potential

Heavy Smartphone users were also found to have reduced early TEP in rPFC reflecting reduced resting state glutamatergic excitability [50]. The momentary change in EEG recorded activity caused by TMS is considered to reflect depolarization of neuronal populations underneath the stimulating coil. TEP is thus associated with resting state excitability of the stimulated brain region, as was previously reported in Alzheimer disease and sleep deprivation [51,52]. This pattern of reduced rPFC excitability in SU resonates well with previous structural and functional imaging studies which found hypoactivity particularly in these brain regions in ADHD patients [53] and in internet and gaming addiction [31]. Abnormalities in rPFC activity have been previously suggested to reflect deficits in a wide range of cognitive capacities [54] such as decision making processes, emotional regulation, executive functioning, working memory, impulsivity and behavioral inhibition. More specifically, the observed correlations with hyperactivity and inattention measures suggest that reduced prefrontal excitability is associated with reduced behavioral inhibition in heavy smartphone users. This association is corroborated by the abovementioned internet addiction and ADHD [53] literature linking right prefrontal hypoactivity with inattention and impulsivity.

The absence of a stable reliable LICI effect and the relative small sample size renders any inferences regarding the effect of usage on neural inhibition premature.

### General limitations

There are several important limitations to this preliminary report which should be addressed in any future attempts to characterize the effects of standard smartphone usage. First, the sample size in the second experimental phase is small and the effect sizes are small-moderate. This limitation stems from the difficulty in recruiting non-smartphone users who were willing to undergo TMS treatment and a longitudinal intervention. This type of control design in adults is currently implausible due to the difficulty in recruitment. Nonetheless, to better encapsulate the behavioral effects of short exposure larger sample size studies are still necessary. Second, the study lacks conclusive evidence regarding the neurobiological changes accounting for the behavioral effects observed. Such rapid deterioration in cognitive functions in healthy participants is likely to be mirrored in specific neuronal abnormalities which were not captured here. Conceivably, longer exposure to the device may result in more apparent associations between behavioral and neural phenomenon. However, unfortunately the majority of our control group has independently upgraded their phone following the study thus no further controlled testing could be performed on this sample. Finally, we could not track actual usage patterns of 'old' mobile phones in nonusers. Hence we cannot rule out the possibility that some were in fact heavy users of 'old' phones. However, we feel confident to assume that on average the usage of such phones was within normal limits and smaller than that of smartphone users.

### Conclusion

For a device that dictates such a large volume of daily functions, the smartphone has gained little research attention to date and so its effects on cognition remain largely undiscovered. This pioneering attempt to reveal some of the cognitive and neurobiological costs of smartphone usage found that smartphone usage rapidly impairs arithmetic numerical processing capacity and increases negative social cognitions, while short-term non-verbal memory function is not significantly affected. Heavy smartphone users also expressed increased impulsivity, impaired attentional functions which correlated with reduced prefrontal neuronal excitability and lastly abnormal inter hemispheric signal propagation. Finally, the frequency of usage in these heavy users predicts the extent of inattention problem they experience.

We suggest that the capacity of the device to capture and harbor attention and gratify immediate rewards induces long lasting effects on a wide range of cognitive abilities. Technological advancements inevitably transform our cognition and neuronal architecture. This rapid process occurs largely without much scientific documentation. This study is a small step in shedding light on these changes while they become fully cemented in our cognition.

## Supporting information

S1 Table Socio-demographic characteristics of phase 1 sample. (DOCX)

S2 Table Socio-demographic characteristics of phase 2 sample. (DOCX)

S1 File Stop Signal EEG and TMS-EEG Protocol. (DOCX)

S1 Fig Induction of long-interval cortical inhibition in the rPFC by paired TMS pulses. (a) Grand average rectified ERPs induced by single pulses (SP) and paired pulses (PP) over the rPFC of SU and NU participants. Note the inhibited paired pulses as compared to single pulses in both groups. (b) LICI in the SU group (29%±5%) was higher on average than that of the NU group (26%±6%) (t(42)<1, p = 0.8). (c) The bottom right panels presents a two dimensional topographical plot of LICI during the time window of 50–150 ms after the test pulse. In lines with previous reports using the same signal processing parameters the points of maximal inhibition appear proximate to but not immediately under the coil location.
(TIF)

S2 Fig Electrophysiological response to single TMS pulses in the rPFC. Grand average rectified ERP plots of early TEP taken from all electrodes under the stimulation coil (FC4,F4,FC6,F6) in the Smartphone users (SU) and nonusers (NU) groups.
(TIF)

## Abbreviations

rPFC: right prefrontal cortex
RMT: resting motor threshold
TEP: TMS evoked potential
LICI: Long-interval cortical inhibition
CAS: concern for appropriateness scale
