# Supplementary material for: Answering the missed call: Initial exploration of cognitive and electrophysiological changes associated with smartphone use and abuse
Source: PLoS One. 2017 Jul 5;12(7):e0180094. doi: 10.1371/journal.pone.0180094 (PMC5497985; doi:10.1371/journal.pone.0180094)
Supplement: S1 Table — (DOCX) [file pone.0180094.s001.docx]

Supplementary Table S1. Socio-demographic characteristics of phase 1 sample.

Table S1. Socio-demographic characteristics of study population in experimental phase 1

|  | Nonuser (NU, n=35) | Heavy users (SU, n=16) |
| --- | --- | --- |
| Age (mean±SD) | 25±3.8 [range: 21-32 years] | 24±2.5 [range: 21-27 years] |
| Gender | 19 females | 9 females |
| Years of Education (mean±SD) | 14.1±1.07 | 13.8±1.58 |
| Main occupation | Student (n=27), research (n=1), IT services (n=1), food industry (n=1) | Student (n=15), teacher (n=1) |
| Socio-economic cluster^*^ (mean±SD) | 7.2±2.16 | 7±2.3 |
| Hours of part time work per week (mean±SD) | 15±6.5 | 14.25±8.1 |
| Laptop computer ownership | 90% | 87.5% |

^*^Socio-economic cluster was based on a governmental 1-10 socio-economic score of participants’ home address.
