# Supplementary material for: Answering the missed call: Initial exploration of cognitive and electrophysiological changes associated with smartphone use and abuse
Source: PLoS One. 2017 Jul 5;12(7):e0180094. doi: 10.1371/journal.pone.0180094 (PMC5497985; doi:10.1371/journal.pone.0180094)
Supplement: S2 Table — (DOCX) [file pone.0180094.s002.docx]

Supplementary Table S2. Socio-demographic characteristics of phase 2 sample.

Table S2. Socio-demographic characteristics of study population in experimental phase 2

|  | Nonusers controls remaining with old phone (NUco, n=14) | Nonusers receiving smartphone (NUsp, n=11) |
| --- | --- | --- |
| Age (mean±SD) | 24.9±2.2 years [range 22-28] | 24.7±2 years [range: 21-29] |
| Gender | 8 females | 7 females |
| Years of Education (mean±SD) | 14.4±0.9 | 14.1±1 |
| Main occupation | Student | Student |
| Socio-economic cluster^*^ (mean±SD) | 7.07±2.11 | 7.2±2.18 |
| Hours of part time work per week (mean±SD) | 14.6±6.9 | 15.1±5.2 |
| Laptop computer ownership | 93% | 91% |

^*^Socio-economic cluster was based on a governmental 1-10 socio-economic score of participants’ home address.
