# Supplementary material for: Answering the missed call: Initial exploration of cognitive and electrophysiological changes associated with smartphone use and abuse
Source: PLoS One. 2017 Jul 5;12(7):e0180094. doi: 10.1371/journal.pone.0180094 (PMC5497985; doi:10.1371/journal.pone.0180094)
Supplement: S1 File — (DOCX) [file pone.0180094.s003.docx]

**Supplementary Information S1. Stop Signal EEG and TMS-EEG Protocol.**

Stop signal task procedure

Participants were required to perform a visual stop signal task (1,2), which is a binary-choice RT task wherein a Go visual stimulus (letter “X” or “O”, positioned in screen center, surrounded by a white square silhouette. Visual angle of the stimulus was 1.755°) guides a motor response. Response keys were counter-balanced between subjects. Participants were instructed to press the response button as quickly and accurately as possible. In a case of failure to respond within 1000 ms, auditory feedback was played.

In 25% of trials participants were required to inhibit their response when a stop signal appeared after the go signal (square turned opaque white). The stop signal delay (SSD) changed through the experiment in a staircase dynamic-tracking manner, depending on the subject’s performance in the preceding ‘stop’ trial. When participants failed to inhibit their response, SSD was consequently lengthened by 50 ms, while in case of inhibition failure SSD was shortened by the same duration. The dynamic tracking pursuit 50% successes in inhibition performance thus is most suitable in the setting of EEG\ERP because it yields sufficient and similar amount of data segments in each performance condition.

The task was programmed in E-Prime Studio version 2.0.10.242 (Psychology Software Tools, Sharpsburg, PA, USA).

TMS-EEG protocol and determination of resting motor threshold

The coil was held tangentially to the skull, over the optimal spot at the right motor cortex to elicit motor evoked potential (MEP) in the Abductor Pollicis Brevis (APB) with the handle pointing backwards/laterally approximately midway between the sagittal and coronal planes. Intensity of pulses was set to 120% of resting motor threshold (RMT) in order to elicit MEPs of around 1 mv amplitude in both the ADM and the FDI. Individual RMTs were determined prior to the experiment as the minimal intensity required to elicit an MEP in around 3 out of 6 single pulses when the hand was fully relaxed. Resting motor threshold (RMT) was roughly identical between NU (72.2%±9.1) and SU (69.8%±9.0) groups (allowing the assumption of equal distribution of the decay artefact in the EEG analysis between groups). After defining the resting motor threshold (RMT), the coil was moved forward 5 cm anterior to the motor spot and 2 cm to the right (over the right PFC). The coil was then locked to the position using adhesive pads attached to a cloth cap (Brainsway, Jerusalem, Israel) which was placed over the EEG cap. Pulses were delivered at 120% of RMT. The stimulation protocol consisted of a series of 5 single pulse followed by a series of 5 paired pulses at 0.2Hz. Paired pulses were interleaved by 100 ms between the conditioning pulse/stimulus (CS) and the test pulse (TS) to induce long-interval cortical Inhibition (LICI). This series was repeated 10 times cumulating at 50 single and 50 paired pulses. A post- report form was used to document any adverse effects of TMS

EEG data preprocessing

**Processing before ICA**

EEG data pre-processing was conducted condition blind. Electrophysiological data was processed offline using EEGlab toolbox for Matlab (1). EEG recordings during TMS were first prepared for Independent Component Analysis (ICA) in the following steps:

1. The first 15 ms after TMS pulse were erased in order to avoid distortion of ICA decomposition by the large amplitude activity evoked by the TMS in this period of time (3). For the long intracortical inhibition (LICI) analysis the first 40 ms TMS pulse were erased as the signal of interest started 50 ms after pulse onset.
2. Segmentation of the data was made with respect to the TMS stimulus (in paired pulse trials segmentation was made in respect to the second (i.e. test) stimulus) such that each segment included a 1000 ms pre-stimulus baseline and a 1000 ms post-stimulus activity.
3. Segments were subsequently manually inspected for removal of noisy channels and noisy segments.
4. All channels were referenced to the average and corrected baseline
5. Segments were baseline corrected to a time window of 500-100 ms prior to stimulation.
6. Data was ICA decomposed first time to remove the exponent artefact and a second time to remove other non-brain components (eye blinks and decay artefact) (number of components removed: NU: 6.26±3.3 SU 6.25±3.6 , NUsp: 6.3 ±2.7 NUco: 7.6±3.3 )
7. Noisy channels were interpolated.
8. Evoked Related Potentials (ERP) for single and paired TMS evoked potential (TEP) were then averaged per participant.
9. Rectified ERP was calculated per participants to derive group mean TEP and assess group differences.

Overall, no significant difference in rejection of trials was found between conditions and groups (Mean trial rejection rates: SU 15%±5.1; NU 16.1%±8.2; NUco baseline 13.7%±5.1; NUsp baseline 14%±5.6; NUco +3 months 13.9%±6.5; NUsp +3 months 18.2%±4.7). Datasets with less than 70 valid trials were discarded from the analysis (TEP: NU, n=1 ;SU, n=1; NUsp, n=1; NUco n=2; LICI: NU, n=2 ;SU, n=3; NUsp, n=1; NUco n=2).

References

1. Logan GD, Cowan WB. On the ability to inhibit thought and action: A theory of an act of control. In: Psychological Review. 1984. p. 295–327.

2. Berger A, Alyagon U, Hadaya H, Atzaba-Poria N, Auerbach JG. Response inhibition in preschoolers at familial risk for attention deficit hyperactivity disorder: A behavioral and electrophysiological stop-signal study. Child Dev. 2013;84(5):1616–32.

3. Rogasch NC, Fitzgerald PB. Assessing cortical network properties using TMS-EEG. Hum Brain Mapp. 2013;34:1652–69.
