# Supplementary figures and images for: Answering the missed call: Initial exploration of cognitive and electrophysiological changes associated with smartphone use and abuse
Source: PLoS One. 2017 Jul 5;12(7):e0180094. doi: 10.1371/journal.pone.0180094 (PMC5497985; doi:10.1371/journal.pone.0180094)

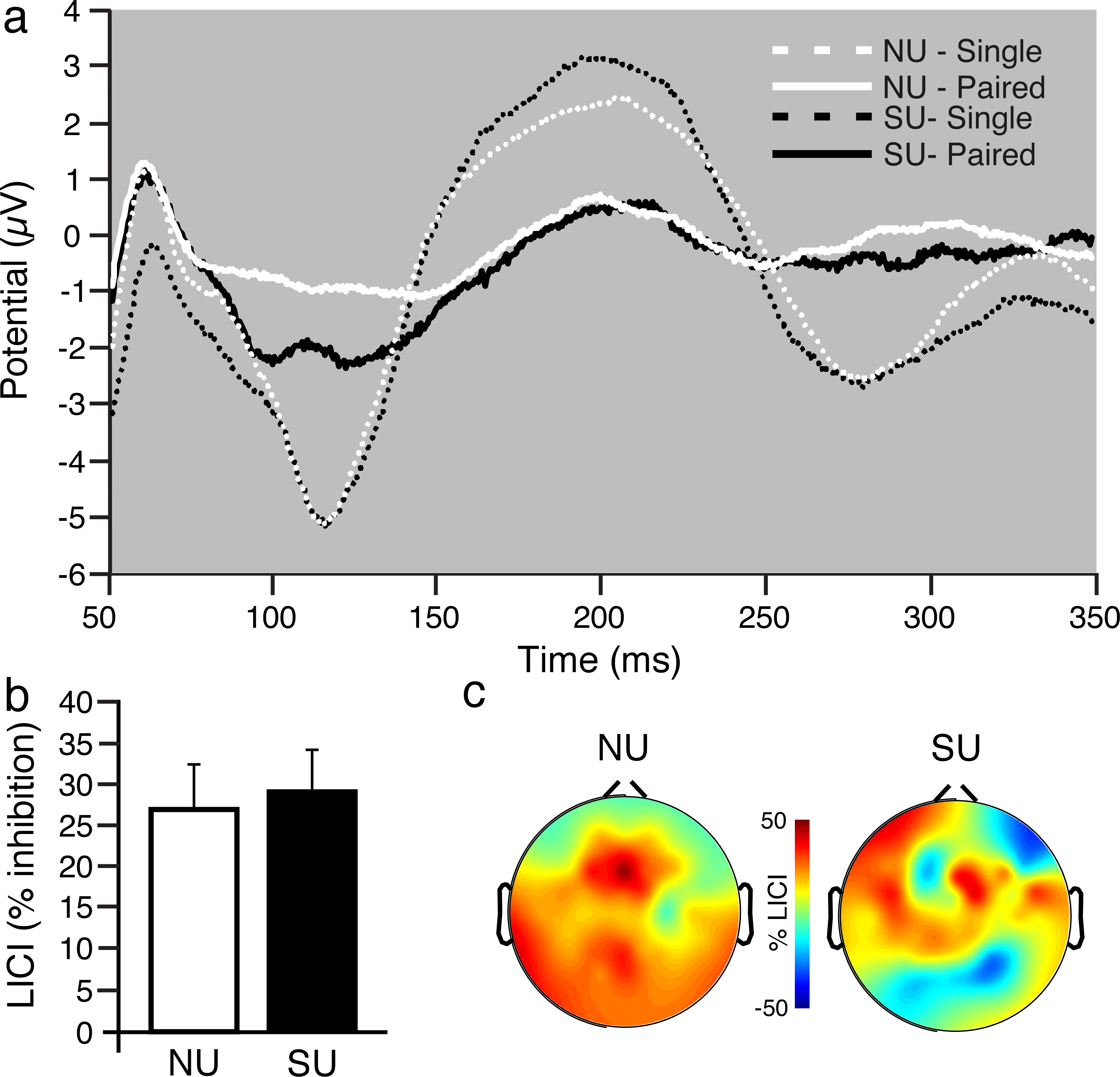

Supplement: S1 Fig — (a) Grand average rectified ERPs induced by single pulses (SP) and paired pulses (PP) over the rPFC of SU and NU participants. Note the inhibited paired pulses as compared to single pulses in both groups. (b) LICI in the SU group (29%±5%) was higher on average than that of the NU group (26%±6%) (t(42)<1, p = 0.8). (c) The bottom right panels presents a two dimensional topographical plot of LICI during the time window of 50–150 ms after the test pulse. In lines with previous reports using the same signal processing parameters the points of maximal inhibition appear proximate to but not immediately under the coil location. (TIF) [file pone.0180094.s004.tif]

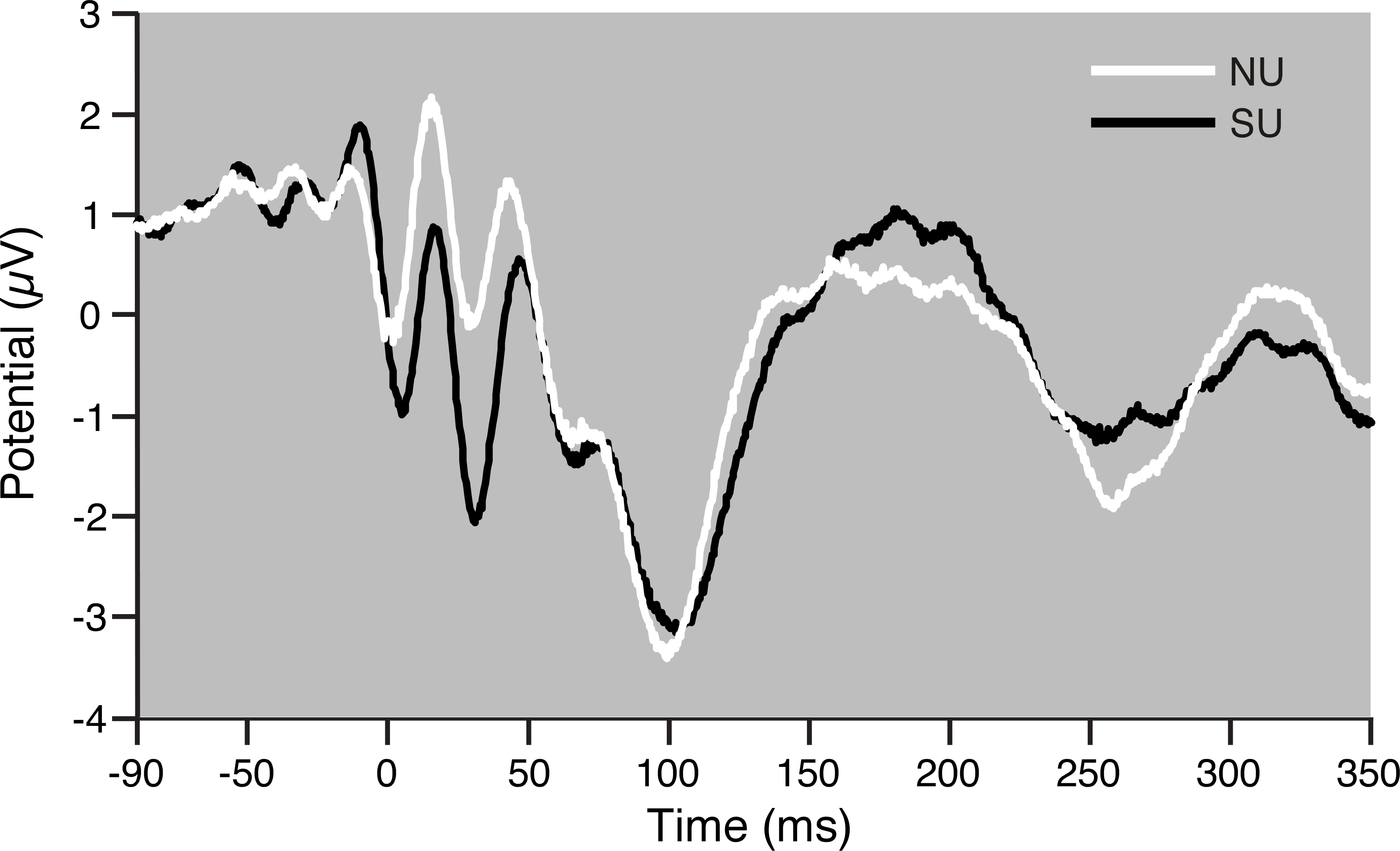

Supplement: S2 Fig — Grand average rectified ERP plots of early TEP taken from all electrodes under the stimulation coil (FC4,F4,FC6,F6) in the Smartphone users (SU) and nonusers (NU) groups. (TIF) [file pone.0180094.s005.tif]
